# Tuning Adipogenic Differentiation in ADSCs by Metformin and Vitamin D: Involvement of miRNAs

**DOI:** 10.3390/ijms21176181

**Published:** 2020-08-27

**Authors:** Sara Cruciani, Giuseppe Garroni, Francesca Balzano, Renzo Pala, Emanuela Bellu, Maria Laura Cossu, Giorgio Carlo Ginesu, Carlo Ventura, Margherita Maioli

**Affiliations:** 1 Department of Biomedical Sciences, University of Sassari, Viale San Pietro 43/B, 07100 Sassari, Italy; sara.cruciani@outlook.com (S.C.); giugarroni21@gmail.com (G.G.); mariafrancesca22@virgilio.it (F.B.); renzopala6@gmail.com (R.P.); ema.bellu@hotmail.it (E.B.); 2 Department of Medical, Surgical and Experimental Sciences, General Surgery Unit 2 “Clinica Chirurgica”, University of Sassari, Viale San Pietro 8, 07100 Sassari, Italy; mlcossu@uniss.it (M.L.C.); ginesugc@uniss.it (G.C.G.); 3 Laboratory of Molecular Biology and Stem Cell Engineering, National Institute of Biostructures and Biosystems-Eldor Lab, Innovation Accelerator, Consiglio Nazionale delle Ricerche, 40129 Bologna, Italy; ventura.vid@gmail.com; 4 Department of Biomedical Sciences, Center for Developmental Biology and Reprogramming (CEDEBIOR), University of Sassari, Viale San Pietro 43/B, 07100 Sassari, Italy; 5 Istituto di Ricerca Genetica e Biomedica, Consiglio Nazionale delle Ricerche (CNR), 09042 Monserrato, Cagliari, Italy

**Keywords:** stem cells, miRNA, cellular mechanisms, gene expression, epigenetic, adipogenesis, conditioned media

## Abstract

Fat tissue represents an important source of adipose-derived stem cells (ADSCs), which can differentiate towards several phenotypes under certain stimuli. Definite molecules as vitamin D are able to influence stem cell fate, acting on the expression of specific genes. In addition, miRNAs are important modulating factors in obesity and numerous diseases. We previously identified specific conditioned media able to commit stem cells towards defined cellular phenotypes. In the present paper, we aimed at evaluating the role of metformin on ADSCs differentiation. In particular, ADSCs were cultured in a specific adipogenic conditioned medium (MD), in the presence of metformin, alone or in combination with vitamin D. Our results showed that the combination of the two compounds is able to counteract the appearance of an adipogenic phenotype, indicating a feedforward regulation on vitamin D metabolism by metformin, acting on CYP27B1 and CYP3A4. We then evaluated the role of specific epigenetic modulating genes and miRNAs in controlling stem cell adipogenesis. The combination of the two molecules was able to influence stem cell fate, by modulating the adipogenic phenotype, suggesting their possible application in clinical practice in counteracting uncontrolled lipogenesis and obesity-related diseases.

## 1. Introduction

Adipose-derived stem cells (ADSCs) are one of the most promising cells for regenerative medicine, due to their higher differentiation potential and easy propagation, as compared to other sources [1,2]. These cells, located in the stromal vascular fraction (SVF) of adipose tissue, exert a central role during adipogenesis, differentiating to produce mature adipocytes [3]. As mesenchymal stem cells (MSCs), they exhibit multipotency features, being able to maintain their undifferentiated state, controlled by the stemness related genes (Oct-4, NANOG, Sox2), or undergo differentiation [4,5].

Peroxisome proliferator-activated receptor γ (PPAR-γ) is a master regulator of adipocyte differentiation. PPAR-γ is required not only during adipogenic differentiation, but also in modulating insulin sensitivity and adipocyte metabolism [6]. In addition, several adipogenic surface markers as activating signal cointegrator-1 (ASC-1) and proton-coupled amino acid transporter (PAT2) have been identified to target white and brown adipocytes and fat depots for therapeutic purposes and obesity treatment [7,8]. Adipose tissue is essential in regulating homeostasis and metabolism of the whole body. Hypertrophy of resident adipocytes leads to uncontrolled pro-inflammatory cytokine secretion, metabolic stress, insulin resistance, cardiovascular disease, and obesity [9]. Vitamin D deficiency is strictly related to obesity and diabetes [10]. This molecule acts directly regulating adipose tissue metabolism, modulating adiponectin expression and the release of pro-inflammatory cytokines [11]. Several isoforms of cytochrome P450 (CYP) enzymes are responsible for its metabolism, in particular, for the 25-hydroxylation (CYP3A4, CYP27A1) and 1,25 hydroxylation (CYP27B1) [12]. A number of causes are involved in the pathogenesis of obesity. In addition, miRNAs have recently emerged as related to numerous metabolic dysfunctions, in particular for adipose tissue, controlling adipogenesis, insulin resistance, and inflammation [13,14]. For example, miR-145 is involved in adipogenic regulation of porcine and bovine preadipocytes [15,16]. An upregulation of miR-148a is associated with the inhibition of lipogenesis and the downregulation of specific adipogenic markers by direct inhibiting Wnt signaling pathway [14,17]. It is known that epigenetic factors, as for example, histone deacetylases (HDACs), are able to modulate adipogenesis, affecting the differentiation of adipocytes and modulating their metabolic features [18]. SIRT1 is required mainly during the early stages of adipogenesis and its upregulation blocks adipogenic differentiation, while increasing the expression of osteoblast markers [19,20]. SIRT2 maintains energy homeostasis by promoting lipolysis and inhibiting adipocyte differentiation by repressing PPAR-γ and FOXO1 deacetylation [21].

Metformin is currently used for the treatment of type II diabetes in obese patients. This molecule has pleiotropic properties, as anti-hyperglycemic, improving insulin sensitivity, inflammation modulation, and inhibition of intracellular lipid accumulation and adipogenesis, especially at specific concentrations [22,23]. Moreover, preadipocyte treatment with metformin suppresses adipocyte differentiation, altering the miRNA profile [24].

Within this context, we recently demonstrated that a combination of different molecules is able to counteract both the molecular program of adipogenesis, as peroxisome proliferator-activated receptor gamma (PPAR-γ) gene expression, as well as the appearance of lipid droplets. In particular, melatonin exerts a synergic effect with vitamin D on ADSCs, controlling adipose differentiation finely tuning osteogenesis and adipogenesis by epigenetic modifications [18,19].

In the present paper, we aimed at evaluating a novel effect of metformin on ADSCs differentiation, in the attempt to modulate adipogenesis, and investigated the activation of CYP involved in vitamin D metabolism. We also assessed whether, during adipogenic differentiation in the presence of metformin, epigenetic mechanisms and miRNAs may have been involved.

## 2. Results

### 2.1. Exposure to Metformin with or without Vitamin D Increases Stem Cell Potency Reducing the Expression of Adipogenic Markers

**Figure 1:**
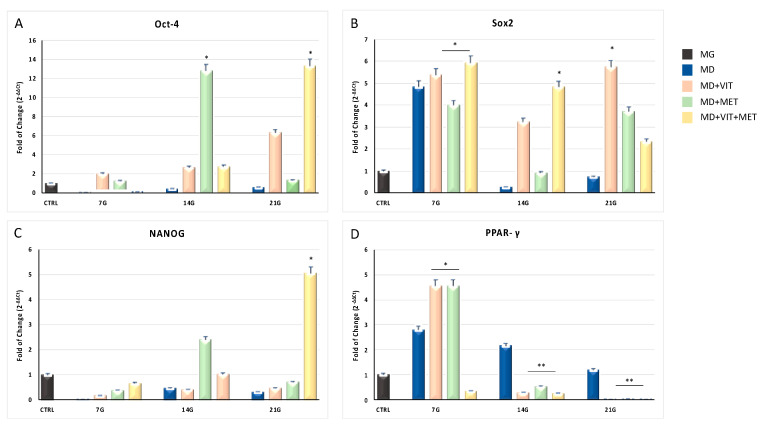
Expression of stemness genes and adipogenic regulating genes. The expression of stemness-related genes Oct-4 (Panel (**A**)), Sox2 (Panel (**B**)), NANOG (Panel (**C**)) and the adipogenic regulating gene PPAR-γ (Panel (**D**)), was evaluated in Adipose-derived stem cells (ADSCs) cultured in: growing medium (MG), adipogenic differentiation medium (MD) (blue bars), or in MD in the presence of vitamin D (orange bars), or in MD in the presence of metformin (green bars), or in MD with metformin plus vitamin D (yellow bars). The mRNA levels for each gene were normalized to glyceraldehyde-3-phosphate-dehydrogenase (GAPDH) and expressed as fold of change (2^−ΔΔCt^) of the mRNA levels observed in undifferentiated control ADSCs (black bars) defined as 1 (mean ± SD; *n* = 6). Data are expressed as mean ± SD referred to the control (* *p* ≤ 0.05; ** *p* ≤ 0.01).

Figure 1 shows the mRNA levels of stemness-related genes in Adipose-derived stem cells (ADSCs) exposed to different conditioned media for 7, 14, and 21 days. As expected, when cells were exposed to the adipogenic differentiation medium (blue bars), a significant downregulation of Oct-4, Sox2, and NANOG could be observed, as compared to control untreated undifferentiated cells at the end of the observation time. Nevertheless, when metformin or vitamin D or both were added to the culturing medium, the expression of all the tested stemness genes was significantly upregulated, reaching expression values significantly higher than control untreated undifferentiated cells (black bars). At the same time, the expression of the main adipogenic orchestrator gene, Peroxisome proliferator-activated receptor γ (PPAR-γ) (Panel D), was significantly downregulated, as compared to cells exposed to MD alone (blue bars), just after 7 days in culture, reaching a minimum when the two molecules were added together in the differentiation medium (yellow bars).

### 2.2. Metformin and Vitamin D Are Able to Modulate the Expression of CYP

**Figure 2:**
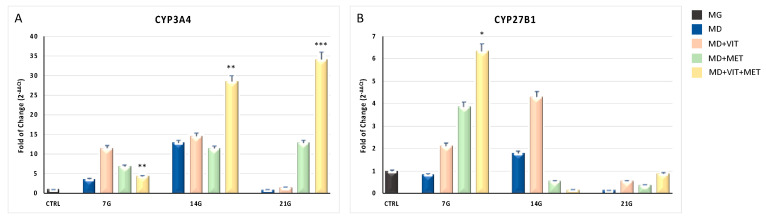
Expression of CYP450. The expression of CYP3A4 (Panel (**A**)) and CYP27B1 (Panel (**B**)) was evaluated in ADSCs cultured in: growing medium (MG), adipogenic differentiation medium (MD) (blue bars), or in MD in the presence of vitamin D (orange bars), or in MD in the presence of metformin (green bars), or in MD with metformin plus vitamin D (yellow bars). The mRNA levels for each gene were normalized to glyceraldehyde-3-phosphate-dehydrogenase (GAPDH) and expressed as fold of change (2^−ΔΔCt^) of the mRNA levels observed in undifferentiated control ADSCs (black bars) defined as 1 (mean ± SD; *n* = 6). Data are expressed as mean ± SD referred to the control (* *p* ≤ 0.05; ** *p* ≤ 0.01; *** *p* ≤ 0.001).

Figure 2 shows the effects of the two molecules on CYP3A4 and CYP27B1 gene expression. CYP3A4 (Panel A) was downregulated after 21 days of the adipogenic differentiation (blue bars), while being significantly upregulated since the first days of culturing ADSCs in the differentiation medium in the presence of metformin (green bars), vitamin D (orange bars), or both (yellow bars). Even CYP27B1 (Panel B) was upregulated after 7 days of exposure, reaching again levels similar to control undifferentiated cells after 21 days of culturing.

### 2.3. Metformin Inhibits Adipogenesis Recruiting Epigenetic Modulating Genes

**Figure 3:**
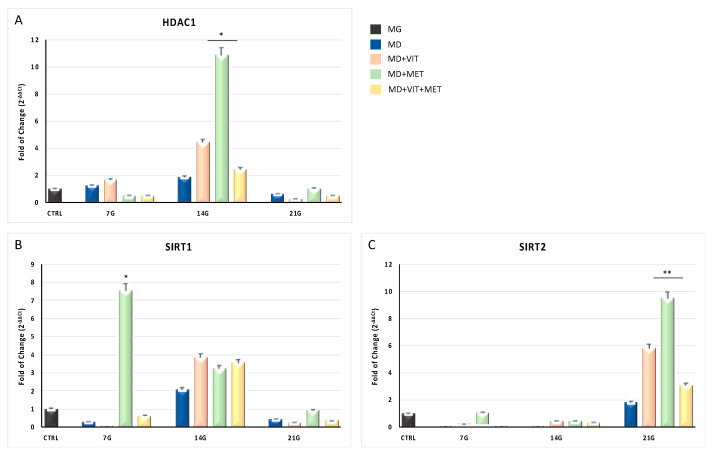
Expression of epigenetic modulating genes. The expression of HDAC1 (Panel (**A**)), SIRT1 (Panel (**B**)), and SIRT2 (Panel (**C**)) was evaluated in ADSCs cultured in: growing medium (MG), adipogenic differentiation medium (MD) (blue bars), or in MD in the presence of vitamin D (orange bars), or in MD in the presence of metformin (green bars), or in MD with metformin plus vitamin D (yellow bars). The mRNA levels for each gene were normalized to glyceraldehyde-3-phosphate-dehydrogenase (GAPDH) and expressed as fold of change (2^−ΔΔCt^) of the mRNA levels observed in undifferentiated control ADSCs (black bar) defined as 1 (mean ± SD; *n* = 6). Data are expressed as mean ± SD referred to the control (* *p* ≤ 0.05; ** *p* ≤ 0.01).

As expected, HDAC1 (Figure 3, Panel A), SIRT1 (Figure 3, Panel B), and SIRT2 (Figure 3, Panel C) are induced during ADSC differentiation. In particular, SIRT2 was significantly increased at the end of the differentiation period (*p* ≤ 0.01) for all culturing conditions, as compared to control untreated undifferentiated cells (black bars). Moreover, SIRT1 and HDAC1 mRNA levels were upregulated (*p* ≤ 0.05) in ADSCs cultured in differentiation medium with or without metformin and/or vitamin D for 7 and 14 days respectively, as compared to control untreated undifferentiated cells.

### 2.4. miRNAs Are Modulated by Metformin

**Figure 4:**
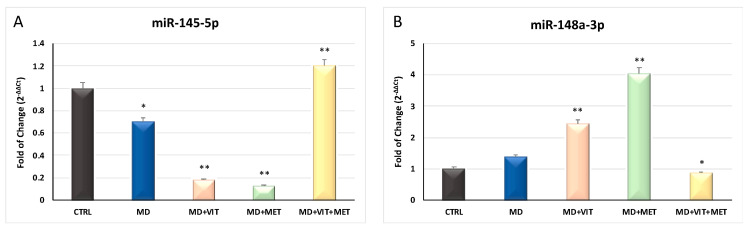
Expression of miRNAs after 21 days of culturing. The expression of miR-145 (Panel (**A**)) and miR-148a (Panel (**B**)) was evaluated in ADSCs cultured in: growing medium (MG), adipogenic differentiation medium (MD) (blue bars), or in MD in the presence of vitamin D (orange bars), or in MD in the presence of metformin (green bars), or in MD with metformin plus vitamin D (yellow bars). The mRNA levels for each gene were normalized to U6snRNA and expressed as fold of change (2^−ΔΔCt^) of the mRNA levels observed in undifferentiated control ADSCs (black bars) defined as 1 (mean ± SD; *n* = 6). Data are expressed as mean ± SD referred to the control (* *p* ≤ 0.05; ** *p* ≤ 0.01).

miRNAs’ levels of expression were evaluated in cells exposed to different conditioned medium after 21 days in culure. As showed in Figure 4, miR-145 (Panel A) was significantly (*p* ≤ 0.01) upregulated only when cells were exposed to both metformin and vitamin D (yellow bars), as compared to control untreated undifferentiated cells (black bars) or MD alone (blue bars). An opposite trend could be observed for miR-148a expression (Panel B), being significantly (*p* ≤ 0.05) downregulated in ADSCs cultured in adipogenic medium in the presence of both metformin and vitamin D (yellow bars) as compared to control untreated undifferentiated cells (black bars).

### 2.5. Metformin Counteract ADSC Adipogenic Differentiation Despite the Presence of a Specific Conditioned Medium

**Figure 5:**
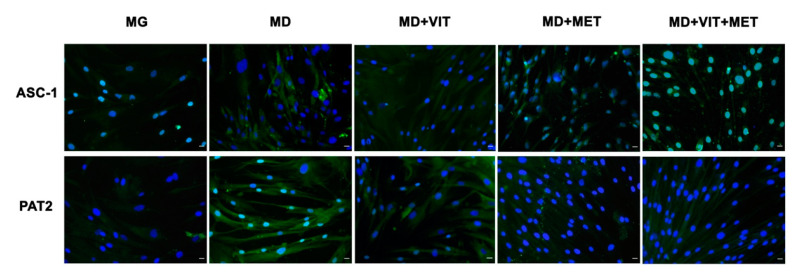
Analysis of adipogenic specific proteins after 21 days in culture. Immunohistochemical analysis of the expression of ASC-1 and PAT2 was assessed in ADSCs cultured in the basic growing medium (MG) or and in the differentiation medium (MD) or the differentiation medium plus vitamin D (MD+VIT) or metformin (MD+MET) or with both vitamin D and metformin (MD+VIT+MET). The figures are representative of different independent experiments. Nuclei are labelled with 4,6-diamidino-2-phenylindole (DAPI, blue). Scale bars: 40 µm.

Immunohistochemical analysis confirmed that during adipogenic differentiation, the presence of metformin alone or together with vitamin D in the differentiation medium inhibits adipogenic differentiation (Figure 5). In particular, the presence of metformin counteracts the expression of the main markers of adipogenesis, activating signal cointegrator-1 (ASC-1) and proton-coupled amino acid transporter (PAT2), being superimposable to control untreated undifferentiated cells. This effect was higher when metformin and vitamin D were contemporarily added to the differentiation medium. On the other hand, ASC-1 and PAT2 are clearly evident in ADSCs committed to the adipogenic phenotype in the presence of the only adipogenic differentiation medium (MD).

### 2.6. The Syngergy between the Two Molecules Ameliorate the Expression of CYP450

**Figure 6:**
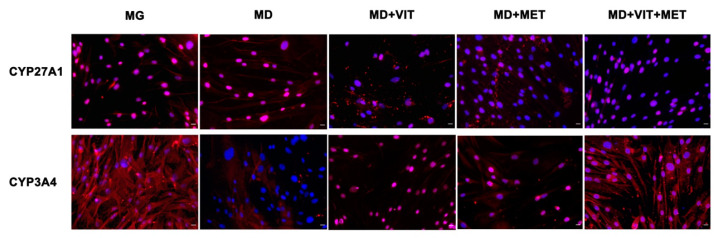
Analysis of CYP450 after 21 days in culture. Immunohistochemical analysis of the expression of CYP27A1 and CYP3A4 was assessed in ADSCs cultured in the basic growing medium (MG) or and in the differentiation medium (MD) or the differentiation medium plus vitamin D (MD+VIT) or metformin (MD+MET) or with both vitamin D and metformin (MD+VIT+MET). The figures are representative of different independent experiments. Nuclei are labelled with 4,6-diamidino-2-phenylindole (DAPI, blue). Scale bars: 40 µm.

During adipogenic differentiation, expression of both CYP3A4 and CYP27A1 could not be detected (Figure 6). On the other hand, the presence of metformin alone or together with vitamin D in the differentiation medium increased the expression of these enzymes involved in the first 25-hydroxylation of vitamin D. In particular for CYP3A4, the levels of expression are clearly evident in ADSCs cultured in the presence of the two molecules, as compared to ADSCs cultured in adipogenic differentiation medium (MD) alone.

### 2.7. Metformin Counteract Fat Droplet Accumulation

**Figure 7:**
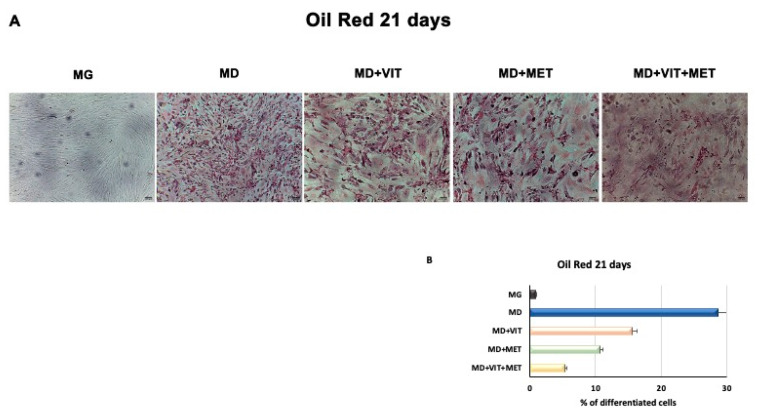
Effect of metformin and vitamin D on lipid accumulation in ADSCs during adipogenic differentiation. (**A**) shows the lipid accumulation in ADSCs cultured in the basic growing medium (MG) or and in the differentiation medium (MD) or the differentiation medium plus vitamin D (MD+VIT) or metformin (MD+MET) or with both vitamin D and metformin (MD+VIT+MET). Scale bar 100 μm. The amount of lipid accumulation and (**B**) was calculated using ImageJ. Data are expressed as mean ± SD.

Figure 7 shows that during adipogenic commitment, the presence of metformin or both metformin and vitamin D in ADSCs exposed to the MD, is able to counteracted intracellular lipid accumulation. As shown, cells cultured in the presence of MD alone showed a higher number of lipid droplets, as compared to contol untreated undifferentiated cells. Metformin was able to inhibit adipogenesis of ADSCs, but this effect was higher when the two molecules were added simultaneously to the MD.

## 3. Discussion

Adipose-derived stem cells (ADSCs) represent a prominent resource for autologous and allogenic transplantation, due to their proliferative and regenerative capabilities [25]. In the adipose tissue, ADSCs represent a fraction of the entire resident population, that can undergo differentiation under specific external stimuli [26]. In the niche in which they reside, ADSCs are responsible for maintaining adipose tissue physiology and its renewal [27]. Moreover, in recent years, different kind of isolation procedures of these cells from the stromal vascular fraction (SVF) have been optimized in order to easily obtain a high yield of stem cells with a higher differentiation potential, as compared to stem cells from other sources [25,28,29,30]. In physiological conditions, ADSCs are involved in the process of adipogenesis, differentiating in pre-adipocytes and mature adipocytes maintaining the homeostasis of adipose tissue [31]. Alterations in ADSC differentiation and in adipose tissue physiology are related to a state of low-grade inflammation and diabetes, leading to obesity [32]. Understanding the molecular mechanisms involved in these alterations could contribute in controlling adipogenesis and its related metabolic complications, as cardiovascular risk. Within this context, we previously demonstrated that the combination of melatonin and vitamin D is able to counteract adipogenic differentiation in ADSCs, reducing cytoplasmatic fatty acid accumulation and maintaining the pluripotency of stem cells [33,34]. In the present paper, we evaluated the effect of metformin and vitamin D, alone or in combination, on ADSC adipogenic differentiation. Metformin is a well-known drug used in the management of obesity, for its capability in reducing body weight in obese patients [35,36]. In addition, vitamin D supplementation has been shown to induce a decrease in weight and body mass index (BMI), reducing the risk of developing type 2 diabetes, cardiovascular diseases, and hypertension [37]. Furthermore, it is demonstrated that obesity is associated with low levels of circulating vitamin D, and with a reduced activity of CYP450 involved in its activation [38,39,40]. To become biologically active, vitamin D must be converted in 1,25-dihydroxyvitamin D (1,25(OH)_2_D_3_). CYP3A4 is considered the most important drug-metabolizing cytochrome P450, with a vitamin D 25-hydroxylase activity [41,42], as well as CYP27A1, both being distributed in various tissues [43]. Once converted in 25-hydroxyvitamin D3, vitamin D must be converted in 1,25(OH)_2_D_3_. This second step involves another 1α- hydroxylase, known as CYP27B1 [44]. Our experiments showed that, during adipogenic differentiation, the presence of metformin within ADSC differentiation medium, alone or in combination with vitamin D, was able to significantly increase the expression of CYP3A4, even at the molecular level, and that of CYP27A1, as compared to cells cultured in the presence of the adipogenic differentiation medium alone (Figure 2, Panel A and Figure 6). We also highlight that the gene expression levels of CYP27B1 were induced by metformin with or without vitamin D during the first 7 days of the differentiation procedure (Figure 2, Panel B). The capability of the two molecules to counteract adipogenic differentiation was further inferred by the significant downregulation of the main markers of the adipogenic phenotype, PPAR- γ (Figure 1, Panel D), ASC-1 and PAT2 (Figure 5), and lipid droplet accumulation (Figure 7). Concomitantly, the maintenance of the multipotent state was assessed by the increased expression of stemness-related genes, Oct-4, Sox2, and NANOG, whose mRNA levels were significantly upregulated along with all the differentiation period when metformin alone or with vitamin D were present (Figure 1, Panels A, B, and C). To this end, the stemness-related genes undergo a significant downregulation when stem cells are committed toward a specific phenotype [45]. We then evaluated the effect of metformin and vitamin D in the modulation of epigenetic genes, HDAC1 and Sirtuins. Other authors previously described that reduced HDAC1, SIRT1, and SIRT2 expression promotes adipogenesis and accumulation of visceral fat in human obesity [46,47]. Here, we show that ADSC exposure to metformin and vitamin D increases the expression of all the above-mentioned epigenetic modulators (Figure 3), suggesting their involvement in blocking adipogenic differentiation. A crucial role in the process of adipogenesis is played by miRNAs. Here, we investigated the expression of miR-145 and miR-148, whose levels were respectively up and downregulated in ADSCs exposed to metformin, together with vitamin D (Figure 4). Other authors previously demonstrated that miR-145 is downregulated during in vivo and in vitro adipogenesis, while its upregulation inhibits adipogenesis by reducing the activity of PI3K/Akt and MAPK signaling pathways [15,16]. Even miR-148a expression is influenced by lipid accumulation. When upregulated, miR-148a promotes adipogenic differentiation, while inhibiting preadipocytes differentiation when downregulated [17,48]. Our results suggest a synergic role of metformin and vitamin D in counteracting adipogenic differentiation, by modulating specific miRNAs, in particular, upregulating the expression of miR-145 and downregulating miR-148. Thus, we could hypothesize that metformin and vitamin D could interfere in an interesting network involving miRNAs and adipogenic orchestrator genes.

## 4. Materials and Methods

### 4.1. Cell Isolation and Treatment

ADSCs were isolated from subcutaneous adipose tissue of human male and female patients (*n* = 6, age = 45 ± 15 years, BMI: 22 ± 3 kg/m^2^), as previously described [16], after signed written informed consent. Briefly, the harvested tissue was washed twice with sterile Dulbecco’s phosphate buffered saline (DPBS) (Euroclone, Milano, Italy) containing 200 U/mL penicillin and 0.1 mg/mL streptomycin (Euroclone, Milano, Italy) and then processed by mechanical and enzymatic digestion in collagenase type I (Gibco Life Technologies, Grand Island, NY, USA). The digested samples were then centrifuged 10 min at 600× *g* and the stromal vascular fraction (SVF) containing ADSCs was resuspended in a basic growing medium composed of Dulbecco’s modified Eagle’s medium (DMEM) (Life Technologies Grand Island, NY, USA) supplemented with 20% fetal bovine serum (FBS) (Life Technologies, Grand Island, NY, USA), 200 mM L-glutamine (Euroclone, Milano, Italy), and 200 U/mL penicillin—0.1 mg/mL streptomycin (Euroclone, Milano, Italy). The culture medium was changed every 3 days and, when cells reached the confluence, they were immunomagnetically selected with a primary monoclonal anti-c/kit (CD117) antibody (MicroBeads, MACS Miltenyi Biotec, Bologna, Italy), and characterized by flow cytometry with primary antibodies directed against CD73, CD90, CD105, CD45, and CD31, as previously described [33]. The resulting ADSCs were used to performed next experiments. A group of cells used as the undifferentiated control was maintained in a basic growing medium (MG). A group of cells was differentiated towards the adipogenic phenotype using a specific conditioned differentiation medium (MD) (StemPro Adipocyte Differentiation Medium, Gibco Life Technologies, Grand Island, NY, USA), as positive control of adipogenic differentiation. Other groups of cells were exposed to MD in combination with other molecules, 10–6 M vitamin D (Sigma Aldrich Chemie GmbH, Munich, Germany) or 5 mM metformin (Sigma Aldrich Chemie GmbH, Munich, Germany) or both (MD+VIT; MD+MET; MD+VIT+MET). All the experiments were performed two times (in three technical replicates), separately for each sample.

### 4.2. Gene Expression Analysis

Gene expression analysis was performed in ADSCs exposed to the above described conditions after 7, 14, and 21 days in culture. Total RNA was extracted using ChargeSwitch Nucleic Acid Purification Technology (Thermo Fisher Scientific, Grand Island, NY, USA) according to the manufacturer’s instructions and quantified by NanoDrop™ One/OneC Microvolume UV-Vis Spectrophotometer (Thermo Fisher Scientific, Grand Island, NY, USA). Then, 1 µg of RNA from each treatment was reverse transcribed using High-Capacity cDNA Reverse Transcription Kit (Thermo Fisher Scientific, Grand Island, NY, USA). Quantitative real-time PCR was performed in triplicate using a CFX Thermal Cycler (Bio-Rad, Hercules, CA, USA) under standard qRT-PCR conditions specified in Platinum^®^ Quantitative PCR SuperMix-UDG Kit protocol (Thermo Fisher Scientific, Grand Island, NY, USA) (50 °C for 2 min, 95 °C for 2 min, and then cycled at 95 °C for 15 s, 55–59 °C for 30 s, and 60 °C for 1 min, for 40 cycles). Target Ct values of each sample were normalized on hGAPDH, considered as a reference gene, whose expression did not show any variation during adipogenesis, although some authors have observed variations in its expression during differentiation [49]. The mRNA levels of ADSCs treated with the previous described condition were expressed as fold of change (2^−ΔΔCt^) of the mRNA levels observed in undifferentiated ADSCs at time 0, define as a control. All primers were designed with Primer3, spanning all exons and highly specific. They are from Invitrogen and are described in Table 1. The qRT-PCR analysis was performed for the following genes: octamer-binding transcription factor 4 (Oct-4); sex determining region Y-box 2 (Sox2); homeobox protein Nanog (NANOG); peroxisome proliferator-activated receptor gamma (PPAR-γ), histone deacetylases class I (HDAC1); histone deacetylases class III or Sirtuins (SIRT 1 and 2); cytochrome P450 family 3 subfamily A member 4 (CYP3A4), and cytochrome P450 family 27 subfamily B member 1 (CYP27B1).

### 4.3. miRNA Expression

The level of expression of hsa-miR-145-5p (miR-145) and hsa-miR-148a-3p (miR-148) was evaluated using a reverse transcription followed by polymerase chain reaction (RT-PCR) using TaqMan^®^ MicroRNA Reverse Transcription Kit, (Thermo Fisher Scientific, Grand Island, NY, USA) as previously described [50]. RNA was extracted from cells using Mirvana MIRNA ISO Kit 10-40ISO (Life Technologies) according to manufacturer’s instructions and are described in Table 2. The raw Ct values for each miRNA and U6snRNA were checked for normal distribution. The Kruskal–Wallis test and Wilcoxon signed-rank test were applied to compare the groups at different times of observation, assuming a *p* value < 0.05 as statistically significant. All the analysis and graphics were performed with the Statistical Package for the Social Sciences (SPSS) software version 13.0 (SPSS Inc., Chicago, IL, USA). 

### 4.4. Immunostaining

ADSCs were cultured for 21 days with different described condition and fixed with 4% of paraformaldehyde (Sigma Aldrich Chemie GmbH, Hamburg, Germany) for 30 min at room temperature. After permeabilization by 0.1% Triton X-100 (Thermo Fisher Scientific, Grand Island, NY, USA) -PBS, cells were washed in PBS three times for 5 min. After washing, ADSCs were incubated with 3% bovine serum albumin (BSA)—0.1% triton X-100 in PBS (Thermo Fisher Scientific, Grand Island, NY, USA) for 30 min and then exposed overnight at 4 °C to the primary anti-mouse monoclonal antibodies directed against activating signal cointegrator-1 (ASC-1) (Santa Cruz Biotechnology, Heidelberg, Germany), proton-coupled amino acid transporter (PAT2) (Santa Cruz Biotechnology), CYP27A1 (Abcam, Cambridge, UK), and CYP3A4 (Abcam, Cambridge, UK). Finally, cells were washed two times in PBS for 5 min and stained at 37 °C for 1 h in the dark with the fluorescence-conjugated goat anti rabbit IgG secondary antibody (Life Technologies, USA) and goat anti mouse IgG secondary antibody (Life Technologies, USA). Nuclei were labelled with 1 µg/mL 4,6-diamidino-2-phenylindole (DAPI) (Thermo Fisher Scientific, Grand Island, NY, USA). All microscopy analyses were performed with a confocal microscope (TCS SP5, Leica, Nussloch, Germany).

### 4.5. Oil Red Staining

To evaluate the lipid droplet accumulation, ADSCs were cultured for 21 days in the above described conditions. After 21 days, cells were fixed for 30 min at RT in 10% formalin, then washed twice in H_2_O and in 60% isopropanol for 5 min. Cells were then incubated in oil red solution for 15 min at RT and washed once with H_2_O to removed excess solution. Adipogenesis was evaluated under light microscopy and analysis of lipid accumulation was performed using image analysis software ImageJ, version 1.8.0 (ImageJ, National Institutes of Health, United States Department of Health and Human Services, USA).

### 4.6. Statistical Analysis

Statistical analysis was performed using Statistical Package for the Social Sciences version 13 software (SPSS Inc., Chicago, IL, USA). The experiments were performed two times with three technical replicates for each treatment. For this study, the distributions of each group variance were evaluated with the non-parametric Kruskal–Wallis rank sum and Wilcoxon signed-rank test, assuming a *p* value < 0.05 as statistically significant.

## 5. Conclusions

Adipogenesis involves several cellular modifications, lipid accumulation, and altered gene expression levels. miRNAs and epigenetic modulators, as HDAC and Sirtuins, promote stem cell differentiation, through modulation of stemness-related genes. Understanding the molecular mechanisms involved in stem cell differentiation processes is a major challenge in the development of regenerative medicine. In the present paper, we exposed ADSCs to a combination of different molecules in the attempt to modulate adipogenic differentiation, and provide novel cues that could be exploited to ameliorate adipogenesis-related metabolic syndrome. Our findings describe for the first time specific miRNAs, as target for metformin and vitamin D, disclosing future application for these molecules in pathological conditions involving miR-145 and miR-148a.

## Figures and Tables

**Table 1:** Primers sequences.

Primer Name	Forward	Reverse
hGAPDH	GAGTCAACGGAATTTGGTCGT	GACAAGCTTCCCGTTCTCAG
Oct-4	GAGGAGTCCCAGGCAATCAA	CATCGGCCTGTGTATATCCC
Sox2	CCGTTCATGTAGGTCTCGGAGCTG	CAACGGCAGCTACAGCTAGATGC
NANOG	CATGAGTGTGGATCCAGCT	CCTGAATAAGCAGATCCAT
PPAR-γ	AATCCGTCTTCATCCACAGG	GTGAAGACCAGCCTCTTTGC
HDAC1	ACTGCTAAAGTATCACCAGAGGG	CACACTTGGCGTGTCCTTTG
SIRT1	CATTTTCCATGGCGCTGAGG	TGCTGGTGGAACAATTCCTGT
SIRT2	TTGCTGAGCTCCTTGGATGG	GGGGAGGGAGCTGTAAGAGA
CYP3A4	TAGCCCAGCAAAGAGCAACA	CAAAAGGCCTCCGGTTTGTG
CYP27B1	CCTGAACCAGACCATGACCC	GAGCCTTTGCCATTCTTCGC

**Table 2:** miRNA accession numbers, symbols, and sequences.

Accession ID Number	Symbol	Sequence
**MIMAT0000437**	hsa-miR-145-5p	GUCCAGUUUUCCCAGGAAUCCCU
**MIMAT0000243**	hsa-miR-148a-3p	UCAGUGCACUACAGAACUUUGU
